# The Changes of Lipid Metabolism in Advanced Renal Cell Carcinoma Patients Treated with Everolimus: A New Pharmacodynamic Marker?

**DOI:** 10.1371/journal.pone.0120427

**Published:** 2015-04-17

**Authors:** Francesco Pantano, Matteo Santoni, Giuseppe Procopio, Mimma Rizzo, Roberto Iacovelli, Camillo Porta, Alessandro Conti, Antonio Lugini, Michele Milella, Luca Galli, Cinzia Ortega, Francesco Maria Guida, Marianna Silletta, Giovanni Schinzari, Elena Verzoni, Daniela Modica, Pierfilippo Crucitti, Annamaria Rauco, Alessandra Felici, Valentina Ballatore, Stefano Cascinu, Giuseppe Tonini, Giacomo Carteni, Antonio Russo, Daniele Santini

**Affiliations:** 1 Department of Medical Oncology, Campus Bio-Medico University of Rome, Via Alvaro del Portillo 200, 00128 Rome, Italy; 2 Department of Medical Oncology, AOU Ospedali Riuniti, Università Politecnica delle Marche, Piazza Roma, 22,60121 Ancona, Italy; 3 Department of Medical Oncology, Fondazione IRCCS Istituto Nazionale dei Tumori, Via Giacomo Venezian, 1, 20133 Milan, Italy; 4 Department of Medical Oncology, Cardarelli Hospital, Via A. Cardarelli 9, 80131, Naples, Italy; 5 Department of Oncology, Oncology Unit B, Sapienza University of Rome, Piazzale Aldo Moro, 5, 00185 Rome, Italy; 6 Department of Medical Oncology, I.R.C.C.S. San Matteo University Hospital Foundation, Viale Camillo Golgi, 19, 27100 Pavia, Italy; 7 Department of Clinical and Specialist Sciences, Urology, Università Politecnica delle Marche, Piazza Roma, 22, 60121, Ancona, Italy; 8 Department of Medical Oncology, San Camillo De Lellis Hospital, Via John Fitzgerald Kennedy, 17, 02100 Rieti, Italy; 9 Department of Medical Oncology, Medical Oncology A, Regina Elena National Cancer Institute, Via Elio Chianesi, 53, 00128 Rome, Italy; 10 Department of Medical Oncology, Azienda Ospedaliera Universitaria Pisana, Via Roma, 67, 56126 Pisa, Italy; 11 Department of Medical Oncology, Institute for Cancer Research & Treatment (IRCC), Strada Provinciale, 142, 10060 Candiolo, Torino, Italy; 12 Department of Medical Oncology, Università Cattolica del Sacro Cuore, Largo Agostino Gemelli, 8, 00168 Rome, Italy; 13 Department of Surgery, Campus Bio-Medico University of Rome, Via Alvaro del Portillo 200, 00128 Rome, Italy; 14 Department of Surgical, Oncological and Oral Sciences, University of Palermo, Palermo, Italy; National Cancer Institute, UNITED STATES

## Abstract

**Background:**

Everolimus is a mammalian target of rapamycin (mTOR) inhibitor approved for the treatment of metastatic renal cell carcinoma (mRCC). We aimed to assess the association between the baseline values and treatmentrelated modifications of total serum cholesterol (C), triglycerides (T), body mass index (BMI), fasting blood glucose level (FBG) and blood pressure (BP) levels and the outcome of patients treated with everolimus for mRCC.

**Methods:**

177 patients were included in this retrospective analysis. Time to progression (TTP), clinical benefit (CB) and overall survival (OS) were evaluated.

**Results:**

Basal BMI was significantly higher in patients who experienced a CB (*p*=0,0145). C,T and C+T raises were significantly associated with baseline BMI (*p*=0.0412, 0.0283 and 0.0001). Median TTP was significantly longer in patients with T raise compared to patients without T (10 vs 6, *p*=0.030), C (8 vs 5, *p*=0.042) and C+T raise (10.9 vs 5.0, *p*=0.003). At the multivariate analysis, only C+T increase was associated with improved TTP (*p*=0.005). T raise (21.0 vs 14.0, *p*=0.002) and C+T increase (21.0 vs 14.0, *p*=0.006) were correlated with improved OS but were not significant at multivariate analysis.

**Conclusion:**

C+T raise is an early predictor for everolimus efficacy for patients with mRCC.

## Introduction

Renal cell carcinoma (RCC) is responsible for about 2–3% of all malignant diseases in adults. The most important feature in the selection of the appropriate therapy is the presence of metastases [1]. The primary treatment is surgery ranging from partial nephrectomy of localized RCCs to cytoreductive nephrectomy in extended tumors with multiple metastases. Then, for advanced, metastatic or recurrent disease a systemic therapy can be administered. In the last years, a better understanding of the role of vascular endothelial growth factor (VEGF) and mammalian target of rapamycin (mTOR) pathways has led to the addition of several agents to the therapeutic landscape of metastatic RCC (mRCC). Most of these compounds inhibit tumor angiogenesis through blockade of VEGF (bevacizumab) or VEGF receptor (VEGFR, sunitinib, sorafenib, pazopanib and axitinib). A second class of agents includes temsirolimus and everolimus, which both exhibit anti-tumor effects through inhibition of the mTOR pathway [2]. Everolimus was approved by the FDA in 2009 for patients with advanced RCC after progression with sunitinib or sorafenib. In the RECORD-1 study, treatment with everolimus prolonged the progression-free survival (PFS) compared to placebo in conjunction with best supportive care in patients who received one VEGFR-Tyrosine-Kinase Inhibitor (TKI) or two prior VEGFR-TKI treatments [3–4]. The mTOR plays an important role in the regulation of cellular function. In RCC, the inactivation of *von Hippel—Lindau tumor-suppressor* gene (*VHL*), a common molecular abnormality in RCC, results in abnormal accumulation of hypoxia inducible factor (HIF), mediated by mTOR, that drives cellular growth and angiogenesis [5–9]. It was demonstrated that mTOR also plays a central role in sensing nutrient availability in the cell and, particularly, in regard to lipid and glucose metabolism [10–12]. Thus, mTOR acts as a controller of both anabolic (lipogenesis, adipogenesis and fatty acid esterification) and catabolic (include lipolysis and β- oxidation) pathways [13]. Under nutrient-poor conditions in a normal cellular environment, downstream Mtor activation is attenuated but, in cancer cells, aberrantly high mTOR activity leads to growth and proliferation, even in nutrient-poor conditions [14–16]. Notably, increases in serum cholesterol, triglyceride, and glucose with mTOR inhibitors have been commonly observed in clinical trials and the incidence in the landmark study RECORD-1 was 50% for hyperglycemia, 71% for hypertriglyceridemia and 76% for Hypercholesterolaemia [3,17–20]. The association of mechanism-based toxicities with improved clinical outcomes in patients with mRCC is a familiar paradigm with other molecularly targeted agents [20]. However, no biomarkers that can predict the efficacy of mTOR inhibitors have been validated. In this retrospective study we therefore hypothesized that the basal values and changes in metabolic assessment before and during therapy with everolimus could reflect the inhibition of mTOR in the cancer cell and could serve as predictors of clinical efficacy of treatment with everolimus in mRCC.

## Patients and Methods

### Study population

The study population consisted of adults (aged 18 years and above) with m RCC treated with everolimus after failure of one or two VEGFR-TKIs. Patients were treated in eleven Italian Institutions between January 2009 and May 2013. Data were retrospectively collected from patients electronic medical records and paper charts. The inclusion criteria were: stage IV renal cell carcinoma histologically confirmed with good or intermediate prognosis in according to Motzer criteria, no previous therapy with mTOR inhibitors; treated with everolimus (10 mg/daily) after failure of one or two VEGFR-TKIs or bevacizumab. Tumor response was evaluated every 8 weeks by clinician assessment and according to the Response Evaluation Criteria in Solid Tumors (RECIST). Total serum cholesterol level (C), triglycerides (T), fasting blood glucose level (FBG) and blood pressure (BP) were measured at baseline (at least two weeks before the start of treatment with everolimus) and were repeated every 4 weeks until the end of treatment with everolimus. Body mass Index (BMI) was evaluated at baseline (before starting everolimus). Only changes higher than 10% were considered real increase from baseline value according to previous report [21].

This study was approved by the Institutional Review Board of Campus Bio-Medico University, Rome, Italy. The procedures to obtain biochemical data and follow-up information are in accordance with the Ethical Principles for Medical Research Involving Human Subjects as formulated in the World Medical Association Declaration of Helsinki (revised in 2008). Patient data were anonymized and de-identified prior to analysis.

### Study objectives

The primary endpoint of this retrospective study was to explore the potential value of change in C or T concentrations and C+T simultaneous raise as predictors of everolimus efficacy on Clinical Benefit (CB) [i.e: best response: Stable Disease (SD) or Partial Response (PR)], Time To Progression (TTP), Overall Survival (OS) in metastatic renal cancer patients. Moreover, we examined the impact of change in other bio-markers linked to Metabolic Syndrome as FBG, and BP on everolimus efficacy. Finally, we evaluated the association between basal BMI pre-everolimus and everolimus activity/efficacy. C, T, FBG and BP were modeled as time-varying covariates over the entire course of treatment.

### Statistical analysis

Baseline patient and disease characteristics as well as changes in serum markers levels were compared by *t* tests for continuous variables (Mann Whitney U test) and χ2 test (Fisher’s exact test) for categorical variables finally correlation analysis were carried out using Spearman’s test. OS and TTP were defined, respectively, as the interval between the start of everolimus to death or last follow-up visit, and as the interval between the start of everolimus to clinical progression or death, or last follow-up visit if not progressed. OS and TTP was determined by Kaplan-Meier product limit method. Cox proportional hazards models were applied to explore patients’ characteristics predictors of TTP and OS in univariate- and multivariable-adjusted analysis using a stepwise selection approach with type I error of 0.05 for model entry and 0.10 for elimination. Additional elimination was applied to identify significant variables. A *p* value <0.05 was considered statistically significant. SPSS software (version 19.00, SPSS, Chicago) was used for statistical analysis.

## Results

### Patient characteristics

One hundred seventy-seven patients were included in this analysis. Patients’ characteristics are summarized in Table 1. Forty-six patients showed a rapidly progressive disease under everolimus treatment [best response: progressive disease (PD)], while 131 achieved a CB from everolimus administration. Table 2 displays the baseline characteristics of the two groups, which were well-balanced, except for basal BMI that was significantly higher in patients who experienced a CBfrom everolimus treatment.

**Table 1:** Patient demographics and disease characteristics.

	Everolimus best response		
BASELINE PATIENT CHARACTERISTICS	PD	SD or PD	*P*
	(N. of patients = 46)	(N. of patients = 131)	
Median baseline cholesterol concentration [mg/dl (95% C.I.)]	180.0 (175.0–218.0)	188.0 (184.0–201.0)	0.389
Median baseline tryglicerides concentration [mg/dl (95% C.I.)]	152.0 (135.0–194.0)	149.0 (148.0–176.0)	0.955
Median baseline fasting glucose concentration [mg/dl (95% C.I.)]	96.0 (96.0–110.0)	94.0 (94.0–105.0)	0.265
Median baseline BMI (95% C.I.)	23.2 (23.1–25.6)	25.9 (25.3–26.7)	0.015
Median baseline systolic blood pressure [mmHg (95% C.I.)]	130.0 (127.0–135.8)	130.0 (130–135.5)	0.517
Median baseline diastolic blood pressure [mmHg (95% C.I.)]	80.0 (76.5–82.2)	80.0 (77.3–81.4)	0.952

**Table 2:** Correlation between baseline metabolic characteristics and the response to everolimus in patients with mRCC.

	PATIENTS WITH BIOMARKER UPRAISING			MEDIAN TIME TO PROGRESSION [Months (95% C.I.)]				MEDIAN OVERALL SURVIVAL			
BIOMARKER	Best response			Patients group		UVA* *P*	MVA** *P*	Patients group		UVA* *P*	MVA** *P*
	SD+RP	PD	Fisher’s exact test *P (RR)*	With biomarker uprising	No biomarker uprising			With biomarker uprising	No biomarker uprising		
	131 pts (%)	46 pts (%)									
**Total serum cholesterol (C)**	76 (58)	15 (33)	0.036 (1.306)	8.0 (4.2–11.8)	5.0 (3.9–6)	0.042	0.083	18.5 (13.9–23.1)	16.0 (12.8–19.1)	0.107	
**Triglycerides (T)**	68 (51)	20 (43)	0.3921 (1.092)	10.0 (6.4–13.5)	6.0 (5.3–6.6)	0.03	0.212	21.0 (46.5–25.5)	14.0 (10.7–17.2)	0.002	0.106
**Cholesterol + Triglycerides (C+T)**	62(47)	11 (23)	0.0056 (1.280)	10.9 (7.7–14.1)	5.0 (4.3–5.7)	0.003	0.005 (HR:0.223)	21.0 (12.4–22.6)	14.0 (10.7–17.2)	0.006	0.743
**Fasting blood glucose (FBG)**	58 (44)	14 (30)	0.1596 (1.148)	7.0 (4.1–9.9)	6.0 (5.2–6.7)	0.328		17.5 (12.4–22.6)	14.5 (12.0–16.9)	0.046	0.26
**Blood pressure (BP)**	17 (13)	7 (15)	0.6131 (0.932)	6.7 (5.4–7.9)	6.0 (4.8–7.1)	0.815		13.0 (11.0–14.9)	17.0 (16.4–19.6)	0.555	

* UVA = Univariate Analysis.

** MVA = Multivariate Analysis.

### Association between baseline C, T, BMI, BP and FBG and the outcome of patients treated with everolimus

**Fig 1:**
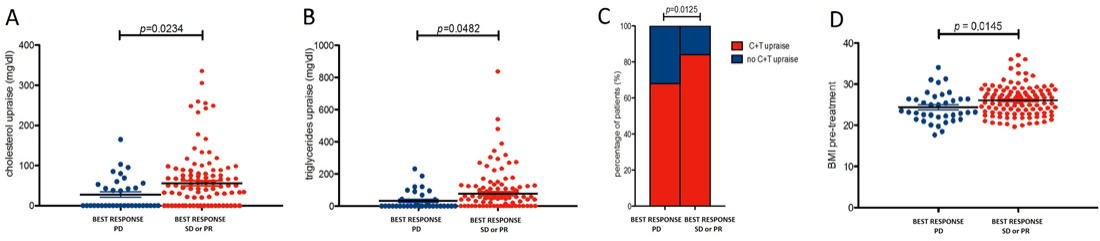
Correlation between change in C (panel A), T (panel B), C+T (panel C) and basal BMI (D) with Clinical Benefit (SD or PR as best response) during everolimus therapy.

**Fig 2:**
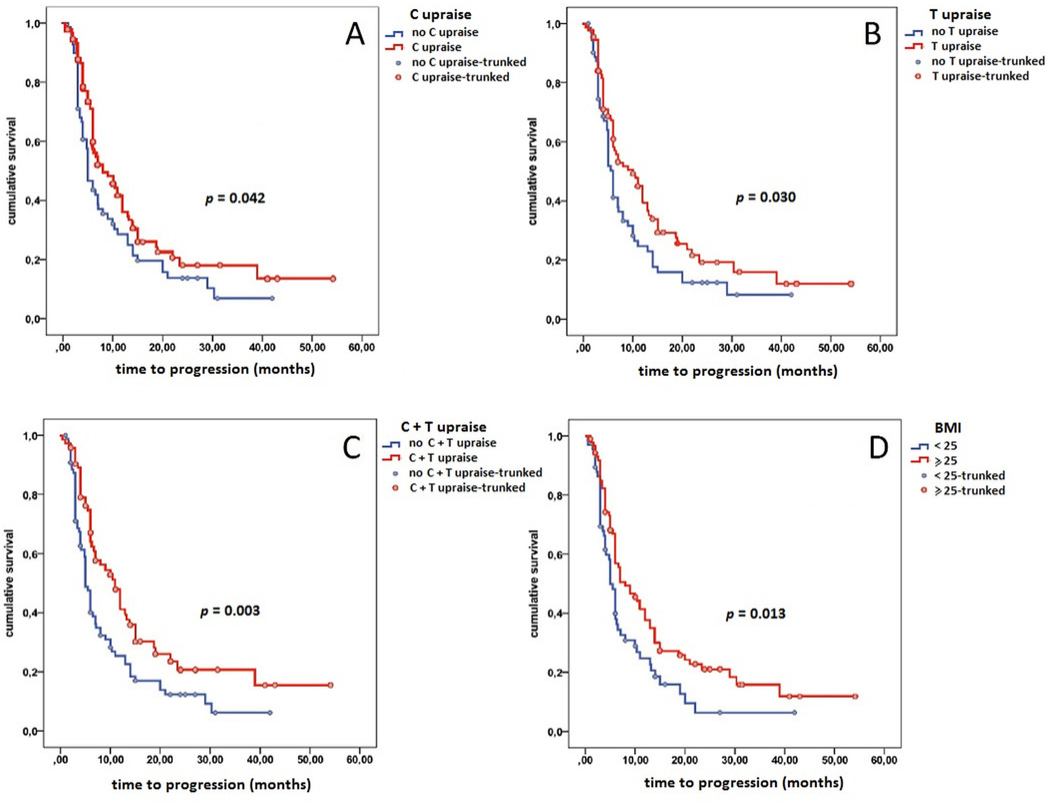
Correlation between change in C (panel A), T (panel B), C+T (panel C) and basal BMI (panel D) with TTP during everolimus therapy.

**Fig 3:**
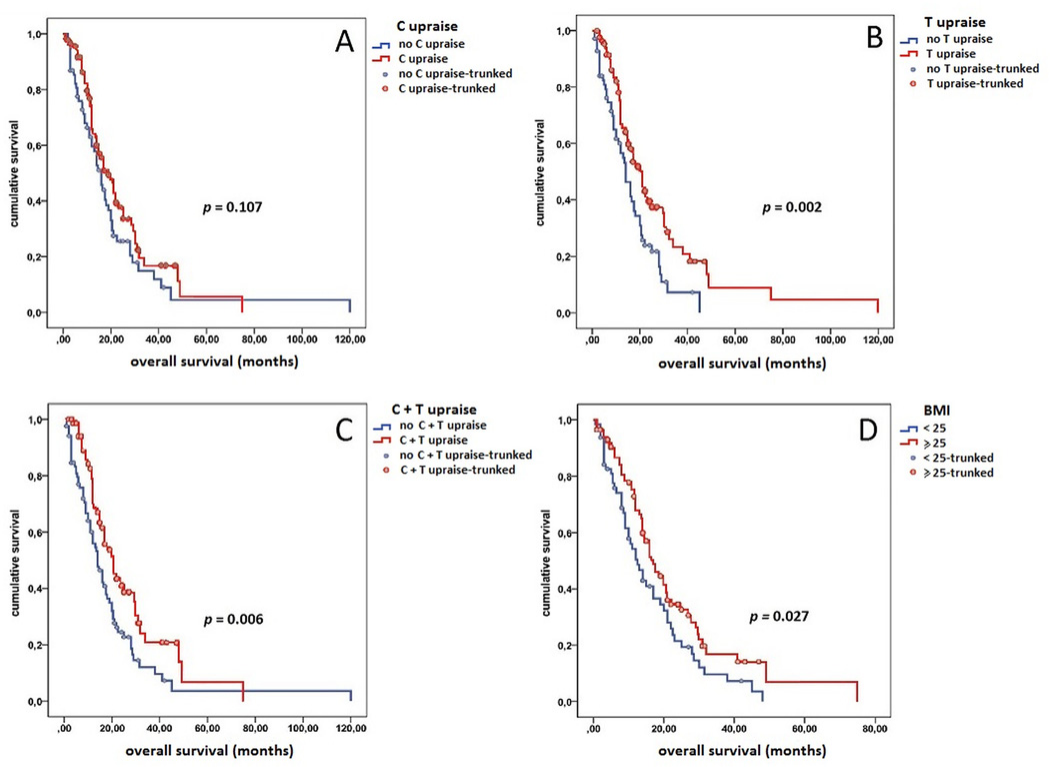
Correlation between change in C (panel A), T (panel B), C+T (panel C) and basal BMI (panel D) with OS during everolimus therapy.

In order to assess the impact of basal biomarkers on clinical outcome we divided the study population into two groups for each parameter according the presence of elevated FBG (cut-off 100 mg/dl sec. International Diabetes Federation (IDF) criteria of metabolic syndrome [22]; median basal value: 95 mg/dl; range: 65–243 mg/dl), BP (cut-off 130 mm/Hg for systolic pressure and 85 mm/Hg for diastolic pressure sec. IDF criteria;22 median basal value: 130 mm/Hg; range 100–160 mm/Hg for systolic BP and 80; range 60–140 mm/Hg for diastolic BP), C (cut-off 200 mg/dL sec. AACE Criteria [23]; median basal value: 187 mg/dl, range: 115–407 mg/dl), T (150 mg/dl sec. IDF criteria [22]; median basal value: 151 mg/dl; range: 56–560 mg/dl) and BMI (cutoff 24.99 sec. WHO Criteria [24]; median BMI: 25.60; range: 17.72–37.11). None of these basal biomarkers correlated with an improved TTP or OS (Table 3). Basal BMI was significantly higher in patients who experienced a CB compared to those with PD as best response during treatment with everolimus 25.91 (95% C.I. 25.34–26.73) vs 23.22 (95% C.I.: 23.11–25.61) *(p* = 0,0145—Fig 1, panel D). Finally, TTP (15.71 vs 9.23 months, *p* = 0.013—Fig 2, panel D) and OS (23.02 vs 16.11 months, *p* = 0.027—Fig 3, panel D) were significantly higher in the 87 patients with elevated basal BMI compared to the 90 patients with normal or low BMI, even if in multivariate analysis this parameter did not demonstrate to be as an independent predictive factor (Table 3).

**Table 3:** Correlation between baseline metabolic characteristics and TTP, and OS of patients treated with everolimus.

		TIME TO PROGRESSION			OVERALL SURVIVAL		
	N. of patients	Median Months (95% C.I.)	UVA*	MVA**	Median Months (95% C.I.)	UVA*	MVA**
	N.(%)		*P*	*P*		*P*	*P*
**PRE-EVEROLIMUS CHOLESTEROL**							
≥200 mg/dl	79 (44.6)	6.3 (4.9–7.7)	0.48		15.0 (10.9–19.1)		
<200 mg/dl	98 (55.4)	6.5 (5.2–7.8)			17.0 (12.8–21.2)	0.25	
**PRE-EVEROLIMUS TRIGLYCERIDES**							
≥150 mg/dl	80 (45.2)	6.0 (4.6–7.5)			14.5 (12.1–16.9)		
<150 mg/dl	97 (54.8)	6.0 (4.9–7.1)	0.95		18.0 (14.0–22.0)	0.86	
**PRE-EVEROLIMUS FASTING BLOOD GLUCOSE (FBG)**							
≥100 mg/dl	78 (44.0)	6.5 (5.1–7.9)			16.5 (12.3–20.7)		
<25 mg/dl	99 (56.0)	6.0 (4.7–7.4)	0.31		16.0 (13.3–18.7)	0.88	
**BASELINE BMI**							
≥25mg/dl	87 (49.1)	8.0 (4.5–11.5)			17 (13.4–20.6)		
<25 mg/dl	90 (50.9)	5.0 (4.2–5.8)	0.01	0.19	12.5 (9.0–16.0)	0.03	0.19
**PRE-EVEROLIMUS SYSTOLIC BLOOD PRESSURE**							
≥120 mmHg	52 (29.3)	7.0 (5.5–8.5)			14.5 (11.5–17.5)		
<120 mmHg	94 (53.1)	5.5 (4.7–6.3)	0.23		14.0 (10.6–17.5)	0.96	
**PRE-EVEROLIMUS DIASTOLIC BLOOD PRESSURE**							
≥80 mmHg	83 (46.9)	7.0 (5.1–8.9)			16.0 (12.9–19.1)		
<80 mmHg	94 (53.1)	6.0 (5.4–6.6)	0.56		14.0 (11.0–17.0)	0.49	

* UVA = Univariate Analysis.

** MVA = Multivariate Analysis.

### Association between baseline BMI and C/T raise

The study population were divided into two groups according to baseline BMI (cut-off 24.99 sec. WHO Criteria [24]; median BMI: 25.60; range 17.73–37.10). Patients with a baseline BMI≥ 25 vs. BMI<25 developed higher C and T raise during everolimus exposure showing respectively a mean C raise of 52.68 mg/dl (95% C.I. 39.55–65.81 mg/dl) vs. 39.54 (95% C.I 23.22–55.86 mg/dl) (*p* = 0.0283) (S1 Fig, panel B) and a mean T raise of 82.59 mg/dl (95% C.I. 56.17–109.22 mg/dl) vs. 39.84 (95% C.I 22.45–57.22 mg/dl) (*p* = 0.0144) (S1 Fig, panel D) showing also a correlation between C (*p* = 0.0412; spearman r: 0.1734) (S2 Fig, panel B) and T (*p* = 0.0283; spearman r: 0.1854) (S2 Fig, panel D) raise and baseline BMI. Moreover patients who developed a C+T raise during treatment with everolimus showed significantly higher mean baseline BMI (28.10, (95% C.I. 24.03–25.69) compared to the patients without C+T upraising (24.80, 95% C.I. 27.19–28.24) (*p* = 0.0001). Interestingly in our cohort baseline BMI was not associated with baseline C values (S1 Fig, panel A; S2 Fig, panel A) and T (S1 Fig, panel C; S2 Fig, panel C).

### C and T raise as a predictor of the outcome of patients treated with everolimus

From the start of everolimus, T increased significantly in 88 patients (50%), with a median increase of 102 mg/dl (range 13–540 mg/dl). The median time to upraising was 60 days (range 15–110 days). C increased significantly in 91 patients (51%) from baseline, with a median increase of 67 mg/dl (range 19–259 mg/dl). The median time to first upraising was 35 days (range: 15–55 days). Finally, C+T raise was registered in 73 patients (41%). The median TTP was significantly longer in patients with T raise compared to patients without T raise (10 vs 6 months, *p* = 0.030) (Fig 2, panel B). Moreover, the median TTP was longer in patients with C raise vs. patients without C raise (8 vs 5 months, *p* = 0.042) (Fig 2, panel A). Finally, Median TTP was 10.9 in patients with C+T raise vs. 5.0 in patients without C+T raise (*p* = 0.003) (Fig 2, panel C). At the multivariate analysis only C+T increase was associated with improved TTP (*p* = 0.005), whereas C or T single raises were not predictors of TTP (Table 2).

As for OS, single T raise (21.0 vs 14.0 months, *p* = 0.002) and C+T increase (21.0 vs 14.0 months, *p* = 0.006) were correlated with improved OS, whereas single C raise was not associated with OS (18.5 vs 16.0 months, *p* = 0.107) (Fig 2, panel A, B and C). However, C+T and T raises were not significant at multivariate analysis (Table 2), probably due to the high percentage (24%) of patients who received a subsequent active treatment (sorafenib) beyond everolimus progression. Furthermore, patients who experienced a CB from the treatment with everolimus showed significantly higher C (*p* = 0.0234) (Fig 1, panel A) and T changes (*p* = 0.0482) (Fig 1, panel B) compared to those who showed PD as best response to everolimus. Finally patients who developed C+T raise were significantly more likely to obtain a CB from everolimus administration (*p* = 0.0125) (Fig 1, panel C).

### Association between changes in BP and FBG and the outcome of patients treated with everolimus

FBG increased significantly in 79 patients (45%) from baseline, with a median increase of 60 mg/dl (range: 10–293 mg/dl). The median time to first upraising was 60 days (range 40–50 days). BP increased significantly in 31 patients (17%), with a median increase of 20 mm/Hg (range: 10–30 mm/Hg) for systolic BP and of 15 mm/Hg (range: 10–30 mm/Hg) for diastolic BP. The median time to first upraising was 39 days (15–79 days). In our study, BP raise was not associated with TTP and OS. In addition, FBG raise correlated with improved OS at univariate (*p* = 0.046), but not at multivariate analysis (*p* = 0.26) (Table 2).

## Discussion

mTOR is a central regulator of cell growth and proliferation in response to growth factor and nutrient signaling. Emerging evidence suggests that mTOR also plays an essential role in sensing nutrient availability in the cell, particularly in regard to lipid and glucose homeostasis [25–27]. The inhibition of mTOR signaling causes global changes in the expression of genes involved in the cell cycle, metabolism, transcription, signal transduction, and many other cellular processes. The mTOR pathway has been implicated in the regulation of sterol regulatory element binding protein (SREBP)-1 and 2 which, respectively, regulate fatty acid and cholesterol biosynthesis. In addition mTOR regulate the expression and the activation state of PPAR-γ and Lipin1. The activation of this complex leads to profound changes in gene expression that ultimately lead to the stimulation of fatty acid uptake, synthesis, esterification, and storage in the newly formed adipose cell [28–30]. RCC have been shown to contain elevated levels of cholesterol esters [31], and some authors have hypothesized that both the enzyme responsible for C ester formation, acyl-coenzyme- A:cholesterol acyl transferase (ACAT), and LDL-mediated uptake may be crucial for RCC progression [32]. However, the complex role of mTOR in regulating the energy balance of RCC tumor cells requires further efforts to better explain the effects of mTOR inhibition on tumor cell metabolism. In the analysis of prognostic factors based on final results of RECORD-1 study, C and T as other bio-markers linked to Metabolic Syndrome were not included [33]. Only in a retrospective analysis of patients treated with temsirolimus in the Global Advanced Renal Cell Carcinoma (ARCC) Trial, longer OS was observed in those who developed hypercholesterolemia during the treatment. The authors proposed the attenuation of SREBP activity as the key factor associated with temsirolimus—induced hypercholesterolemia [21]. However, Wang et al. showed that many functions of SREBP-2 are dependent upon mTOR complex 1 (TORC1) but resistant to rapamycin [34]. In addition, rapamycin has different cell type-specific effects on SREBP-2 processing and the expression of 3- hydroxy-3-methyl-glutaryl-CoA reductase (HMGCR), which is the rate limiting step in cholesterol biosynthesis [35–37]. At this regard, Sharpe and colleagues found that SREBP-2 activation and HMGCR are unaffected by rapamycin treatment. In this study, rapamycin induced a decrease in LDL-receptor gene expression independently of SREBP-2 [37]. However, the evidence that everolimus, still caused hyperlipidemia in LDL receptor-null mice [38] suggest that a decrease in LDL-receptor expression is unlikely to be the only factor that contributes to hyperlipidemic effects seen in patients treated with mTOR inhibitors. On the other hand, Cho et at. Hypothesized that hyperlipidemia during treatment with mTOR inhibitors might be an epiphenomenon associated with slowed tumor growth rather than as a marker of drug efficacy [39]. In our study, we first demonstrate that increased C+T levels, unlike single C or T raises, during treatment with everolimus are significantly associated with improved TTP in patients with mRCC treated with second or third-line everolimus after VEGFR-TKI therapy. Moreover, baseline BMI was associated with C, T and C+T raise but not with their basal values, thus suggesting that high baseline BMI may contribute to the biological mechanisms involved in T and C raise during treatment with everolimus and open new perspectives on the role of BMI and lipid metabolism in cancer progression, actually linked only with risk of RCC development [40–41]. However, there are some limitations to this study. First, this is a retrospective study, which is susceptible to bias in data selection and analysis. The total number of patients analyzed is relatively small. Also, metabolic assessment modifications can be influenced by concurrent drugs that cannot be accounted for in this study. Despite these limitations, our study suggests that changes in C and T levels may be associated with TTP of patients treated with everolimus for mRCC. Patients with an early increase of C and T should be closely monitored for the higher risk of disease progression. Moreover changes in C and T levels may play a pivotal role as pharmacodynamic biomarker in phase I and II studies for the development of next-generation mTOR inhibitors. This role will become quite relevant as everolimus and other mTOR inhibitors are next to be used in various malignancies [42–44].

Prospective studies are needed to assess the potential role of C+T raise and basal BMI value in guiding treatment decisions, patient selection, and clinical trials design.

## Supporting Information

S1 Fig Mann Whitney test analysis and C (panel A and B) or T (panel C and D) raise according to baseline BMI status. (TIF)

S2 Fig Correlation between baseline BMI and C (panel A and B) or T (panel C and D) raise by Spearman rank test. (TIF)
