# Supplementary figures and images for: The Changes of Lipid Metabolism in Advanced Renal Cell Carcinoma Patients Treated with Everolimus: A New Pharmacodynamic Marker?
Source: PLoS One. 2015 Apr 17;10(4):e0120427. doi: 10.1371/journal.pone.0120427 (PMC4401714; doi:10.1371/journal.pone.0120427)

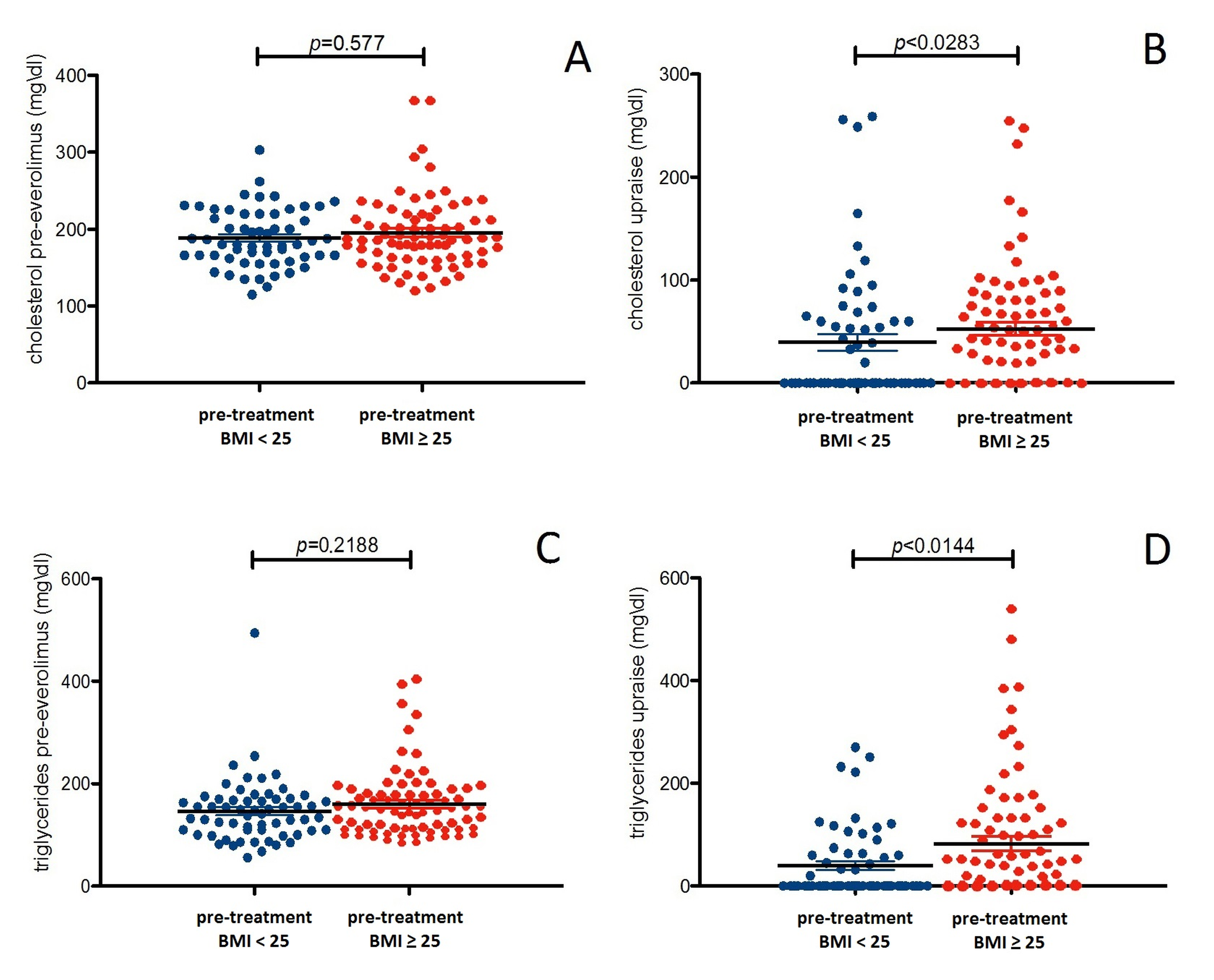

Supplement: S1 Fig — (TIF) [file pone.0120427.s001.tif]

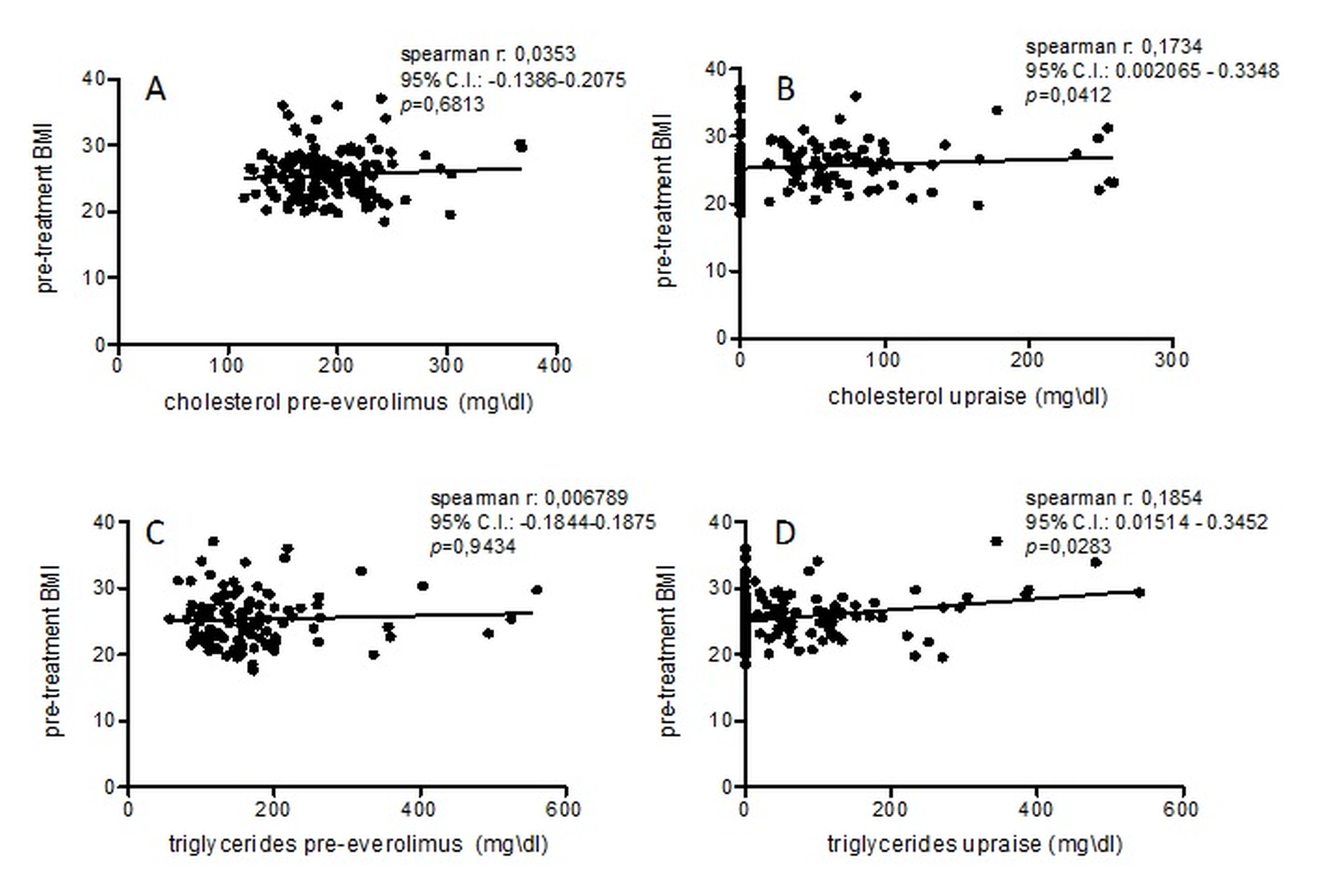

Supplement: S2 Fig — (TIF) [file pone.0120427.s002.tif]
